# Need for better adherence to optimal incubation temperature for quality laboratory diagnostics and antibiotic resistance monitoring

**DOI:** 10.4102/ajlm.v7i2.789

**Published:** 2018-12-06

**Authors:** Cristina Gutierrez, Akos Somoskovi, Kris Natarajan, David Bell

**Affiliations:** 1 Independent consultant, Vigo, Spain; 2 Global Health Technologies, Global Good Fund, Intellectual Ventures Laboratory, Bellevue, Washington, United States

In the omics era, incubation of human specimens and bacterial cultures continues to be the cornerstone for detection, identification and drug susceptibility testing (DST) of bacterial pathogens. Accurate results require bacterial incubation under optimal physicochemical conditions. Temperature is a key physicochemical factor that affects the bacterial environment, making incubators indispensable in clinical laboratories. Human pathogens generally multiply best at temperatures similar to those of the human host (35°C – 37°C). Biochemical tests for identification in pure isolates are recommended to be performed at 36°C ± 2°C, and DST at 35°C± 1°C.^1,2,3,4^ For *Mycobacterium tuberculosis* complex and most nontuberculous mycobacteria, the recommended temperature is 37°C in both cases.

**FIGURE 1:**
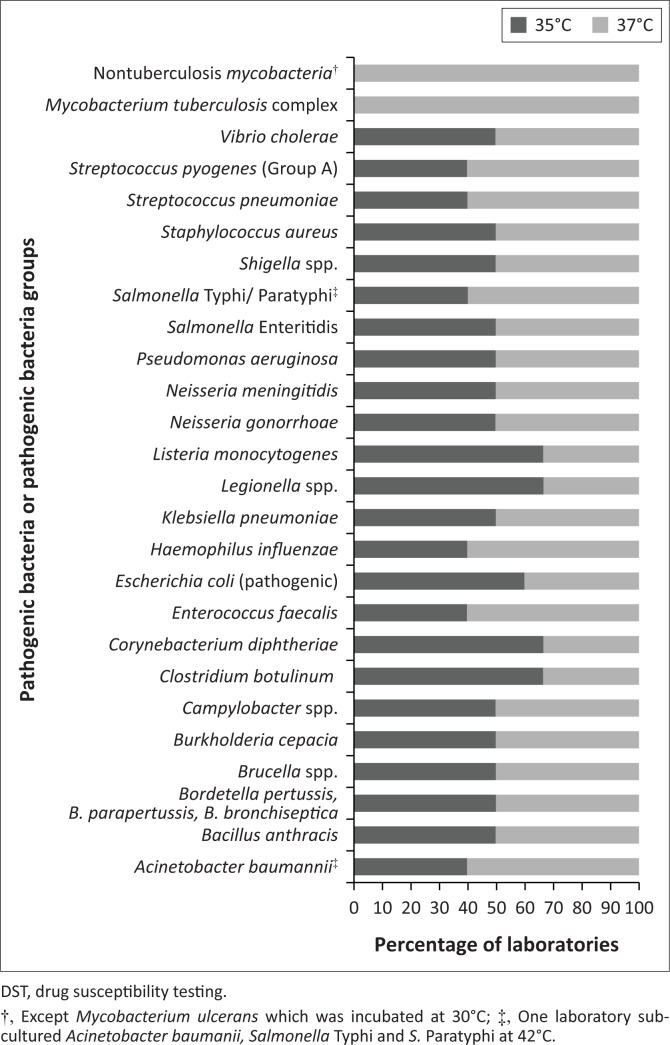
Incubation temperature in 12 laboratories from seven countries for isolation, subculture and DST of 26 pathogenic bacteria or pathogenic bacteria groups frequently isolated from clinical specimens.

In a recent survey to determine the incubator requirements for clinical microbiology laboratories in resource-limited countries, we analysed several parameters including the temperatures used to incubate clinical specimens for primary bacterial isolation, replicating bacterial cultures and performing DST. The data were reported in a self-administered questionnaire conducted from April 2017 to June 2017 by 12 laboratories from three countries in Africa (Cameroon, Ivory Coast and Madagascar), three countries in the Americas (Haiti, Guyana and Bolivia) and one country in Asia (Bangladesh). The clinical specimens considered were sputum, pharyngeal or nasopharyngeal swabs, stools, urethral or vaginal swabs, skin, pus, urine, lymph nodes, blood, bone marrow, cerebrospinal fluid and other normally sterile body fluids. The analysed incubation temperatures referred to 26 pathogenic bacteria or pathogenic bacteria groups frequently isolated from clinical specimens (Figure 1).

Laboratories working with mycobacteria incubated both specimens and isolates at the appropriate temperature of 37°C for *M. tuberculosis* complex and nontuberculous mycobacteria, or at 30°C in the case of *Mycobacterium ulcerans*.

To target the other 24 bacteria or groups of bacteria, specimens were incubated for primary isolation at either 35°C or 37°C with a tendency towards 37°C. Laboratories employed the same distribution of temperatures for sub-culturing isolates and performing DST, with the exception of one laboratory sub-culturing *Acinetobacter baumanii, Salmonella typhi* and *Salmonella paratyphi* at 42°C. The reported temperature fluctuation during incubation was ± 2°C for 55% of the incubators, ± 1°C for 29%, and ± 3°C for 16%.

Clinical laboratories are often faced with the need to grow a priori unidentified bacteria and potentially polybacterial specimens from non-sterile sites. To best support recovery of bacterial pathogens, as well as biochemical testing and DST incubation requirements, a temperature of 35°C is likely ideal. At 35°C, most human bacterial pathogens with differing optimum growth temperature will grow reliably, although colonies may appear small or require additional incubation due to their slower growth rate.^5^ DST is recommended to be performed at 35°C ± 1°C.^3,4^ The use of 37°C by most laboratories puts the reliability of DST results in question. Incubation at 37°C also risks fluctuations to dying off temperatures.

Our field survey revealed fundamental failures regarding basic and easy-to-control requirements of incubation that could negatively impact DST, and therefore the battle against antimicrobial resistance. It is difficult to assess the real magnitude of the problem, because this study relied on self-reported laboratory procedures, which may be subject to bias. With the growing importance of identifying isolates and trends of antibiotic-resistant human pathogens, it is alarming that the simplest global recommendations fail to reach the workplace. More rigorous attention must be paid to bacterial incubation, including DST requirements, through the institution of evidence-based protocols and use of quality instruments that can provide accurate temperature levels, as well as operating under appropriate quality assurance practices and with stricter adherence to existing guidelines.
